# Magnetic control of soft microrobots near step-out frequency: Characterization and analysis

**DOI:** 10.1016/j.csbj.2024.08.022

**Published:** 2024-08-30

**Authors:** Zihan Wang, Wenjian Li, Anke Klingner, Yutao Pei, Sarthak Misra, Islam S.M. Khalil

**Affiliations:** a Department of Biomaterials and Biomedical Technology, University of Groningen and University Medical Center Groningen, Groningen, 9713 GZ, the Netherlands; b Department of Advanced Production Engineering, Engineering and Technology Institute Groningen, University of Groningen, Groningen, 9747 AG, the Netherlands; c Department of Physics, The German University in Cairo, New Cairo, 11835, Egypt; d Department of Biomechanical Engineering, University of Twente, Enschede, 7500 AE, the Netherlands; e RAM—Robotics and Mechatronics, University of Twente, Enschede, 7500 AE, the Netherlands

**Keywords:** Magnetic actuation, Materials characterization, Soft sperm-like microrobots, Step-out frequency, Theoretical model

## Abstract

Magnetically actuated soft microrobots hold promise for biomedical applications that necessitate precise control and adaptability in complex environments. These microrobots can be accurately steered below their step-out frequencies where they exhibit synchronized motion with external magnetic fields. However, the step-out frequencies of soft microrobots have not been investigated yet, as opposed to their rigid counterparts. In this work, we develop an analytic model from the magneto-elastohydrodynamics to establish the relationship between the step-out frequency of soft sperm-like microrobots and their magnetic properties, geometry, wave patterns, and the viscosity of the surrounding medium. We fabricate soft sperm-like microrobots using electrospinning and assess their swimming abilities in mediums with varying viscosities under an oscillating magnetic field. We observe slight variations in wave patterns of the sperm-like microrobots as the actuation frequency changes. Our theoretical model, which analyzes these wave patterns observed without exceeding the step-out threshold, quantitatively agrees with the experimentally measured step-out frequencies. By accurately predicting the step-out frequency, the proposed model lays a foundation for achieving precise control over individual soft microrobots and enabling selective control within a swarm when executing biomedical tasks.

## Highlights

• Our proposed model can accurately predict the step-out frequency of soft microrobots.
• The magnetic torque on the microrobot's head induces the traveling waves along its tail.
• Wave patterns change slightly with actuation frequencies below the step-out frequency.
• Increased viscosity of the medium results in a reduced step-out frequency.

## Graphical abstract

**Figure:**
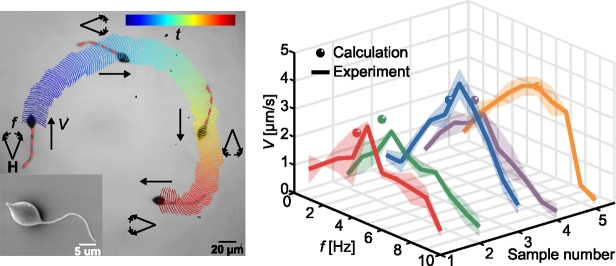

## 1 Introduction

Microrobots actuated by external fields are gaining attention owing to their ability to navigate hard-to-reach positions within the human body. This capability can potentially enhance the precision and dexterity of current surgical procedures [1]. Among a variety of actuation manners, such as light [2], magnetic [3], electric [4], acoustic [5], and thermal [6], magnetic actuation has been extensively studied due to its nontoxic and real-time properties. In recent decades, proof-of-concept studies on magnetic microrobots have been carried out to demonstrate their potential for various biomedical applications, such as multimodal locomotion in nonideal environments [7], enhanced targeted drug delivery [8], and macrophage polarization [9]. Apart from energy sources, the body configurations of microrobots must break time-reversibility to enable movement at low-Reynolds-number flows. Spermatozoa, which achieve propulsion by undulating their soft and flexible flagellum, have inspired the design of mobile microrobots. Magnetically actuated sperm-like microrobots have emerged as promising candidates for executing biomedical tasks within confined and intricate spaces of the body's natural pathways [10].

Magnetically actuated sperm-like microrobots encompass sperm-inspired, sperm-driven, and sperm-templated microrobots. Various microfabrication methods have been proposed to develop these microrobots. Sperm-inspired microrobots are designed using biomimetic principles to imitate the flagellar beating of spermatozoa. For instance, Dreyfus et al. [11] have attached magnetic particles to DNA through the biotin-streptavidin interaction, forming a flexible magnetic filament. When subjected to an oscillating magnetic field, this filament exhibits whip-like deformations and generates propulsive thrust. Building on these principles, the bulk fabrication of sperm-inspired microrobots has been achieved using photolithography and electrospinning [12], [13]. Sperm-driven microrobots can move forward by utilizing the innate motility of spermatozoa. Magdanz et al. [14] have fabricated the spermbot, which consists of a sperm cell entrapped in a magnetic microtube. The incorporation of magnetic microtubes allows for the control of sperm cells' swimming directions under a magnetic field. Furthermore, by fabricating the microtube with a thermo-responsive polymer, the entrapped sperm cells can be released by elevating the temperature [15]. Such a gentle release method keeps the sperm cells intact, which is crucial for assisted fertilization [16], [17]. Sperm-template microrobots refer to the biohybrid microrobots constructed using sperm cells as templates. IRONSperm comprises a sperm cell and rice grain-shaped magnetic nanoparticles via electrostatic self-assembly [18]. In contrast to the spermbot, sperm cells are immotile after binding to magnetic nanoparticles. The IRONSperm does not rely on the ATP consumed by spermatozoa to undulate its flagellum. Instead, its power is provided by external magnetic fields, thus ensuring its long-term locomotion. Such persistent locomotion is the prerequisite for magnetic microrobots intended for minimally invasive medicine, while precise wireless control is equally essential [19]. Wireless control of magnetic microrobots is typically achieved below their step-out frequencies, where they exhibit synchronized motion with the field. This synchronization is crucial for biomedical applications as it enables precise navigation and predictable behavior of the microrobot, minimizing the risk of uncontrolled movement that could cause damage to tissues or cells.

The step-out frequencies of magnetic microrobots are influenced by various factors, such as their magnetization, geometries, field strength, and viscosity of the surrounding medium. Discrepancies in the step-out frequencies lead to distinct responses of magnetic microrobots to the same applied magnetic field [20]. This characteristic can be leveraged as a general method for selective control in multi-microrobot systems. Mahoney et al. [21] have theoretically studied the swimming performance of rotating magnetic microrobots constructed with either a permanent magnet or a soft ferromagnet. By operating both microrobots above their step-out frequencies, the researchers have controlled the velocity ratio of the two geometrically identical but magnetically distinct rolling microrobots, thus steering them to move along different paths. In addition to magnetization, the surface wettability of microrobots impacts their step-out frequency, as it affects the viscous drag force exerted by the surrounding medium. Wang et al. have fabricated a swarm of artificial bacterial flagella (ABFs) that are geometrically and magnetically identical but differ in the step-out frequencies due to variations in surface wettability. Selective control of this ABFs swarm can be accomplished by operating at a frequency below the step-out frequency of the selected group yet above that of the other [22]. Compared with the rigid microrobots above, soft microrobots, capable of substantial and compliant deformation, are more adaptive to changing physical and chemical conditions within the body [23]. However, their flexible characteristic introduces complex interactions with external environments, including the applied magnetic field and the surrounding medium. These combined interactions have not been fully explored, limiting our understanding of the swimming behavior and control mechanisms of soft microrobots.

**Fig. 1:**
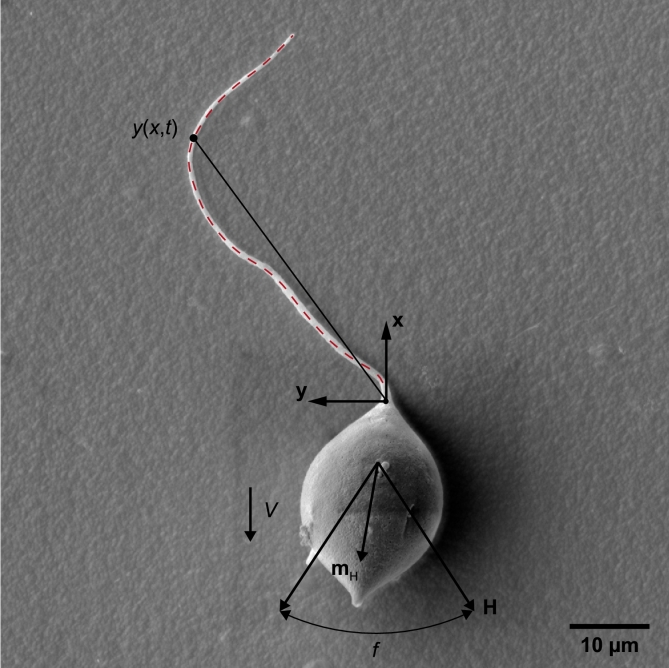
Model of a magnetically actuated soft microrobot. When subjected to an oscillating magnetic field, denoted as **H**, with an actuation frequency of *f*, the head of the microrobot becomes magnetized toward **m**_H_ and aligns with the field. The oscillation of the head drives the tail's deformation, *y*(*x*,*t*), as indicated by the red dashed line. Traveling waves propagate along the tail, generating a propulsive trust that propels the head to swim at a velocity of *V*.

In this work, we propose a theoretical model to study the step-out frequency of magnetically actuated soft microrobots. The step-out frequency is reached when the equilibrium between interactions with the magnetic field and the fluid medium is established. To validate our model, we fabricate soft sperm-like microrobots using electrospinning and sonication cutting (Fig. 1) and investigate their step-out frequencies under an oscillating magnetic field. The effect of the medium's viscosity on the step-out frequencies of these microrobots is assessed by performing swimming tests in deionized (DI) water and Methyl Cellulose (MC) solutions. We characterize their geometries and magnetization using a scanning electron microscope (SEM) and a vibrating sample magnetometer (VSM), respectively, to elucidate the effects of magnetism and fluid dynamics. Energy-dispersive X-ray spectroscopy (EDS) elemental mapping results indicate that magnetic nanoparticles are primarily distributed in the head of the sperm-like microrobot. Based on this experimental observation, we exclusively consider the magnetic torque on the head and develop a magneto-elastohydrodynamic model. This model elaborates the relationship between the step-out frequencies of the sperm-like microrobots and their magnetization, geometries, wave patterns, and the viscosity of the medium. Our methodology combines an established model for calculating the magnetic torque on the microrobot with an elastohydrodynamic model extensively used in studies of natural sperm cells. This integration allows us to investigate the interplay between magnetism, structural flexibility, and fluid dynamics, thereby advancing our understanding of the swimming performance of sperm-like microrobots.

## 2 Material and methods

### 2.1 Preparation of polymer solution for electrospinning

The polymer solution was prepared by mixing dimethylformamide (DMF) (227056-1L, anhydrous, 99.8%, Sigma-Aldrich, The Netherlands)) with a combination of Fe_3_O_4_ nanoparticles (637106-25G, nanopowder, 50-100 nm particle size (SEM), 97% trace metals basis, Sigma-Aldrich, The Netherlands) and polystyrene (430102-1KG, Mw 192,000, Sigma-Aldrich, The Netherlands) at a weight/volume (w/v) ratio of 25%. The mass ratio between Fe_3_O_4_ and polystyrene was 1:2. The detailed procedures are described below. First, 2.5 g polystyrene beads were dissolved in DMF with magnetic stirring for 3 h. Subsequently, 1.25 g Fe_3_O_4_ nanoparticles were dispersed in the solution using an ultrasonic bath for 5 min. Finally, the polymer solution was left on a roller mixer overnight to ensure a uniform blend.

### 2.2 Fabrication of soft sperm-like microrobots

The prepared polymer solution was loaded into a syringe and subsequently mounted on a syringe pump. Operating at the pre-programmed flow rate of 1.2 mL/h, the solution was pumped out, forming a positively charged droplet under a high voltage of 15 kV. The electrostatic repulsion among the surface charges caused the droplet to deform into a Taylor cone at the nozzle tip. As the charged jet was stretched into a slender filament from the cone, it rapidly solidified under the high voltage and finally deposited onto the grounded collector [24]. A sonicator (VCX 130, Sonics & Materials, Inc., USA) generates ultrasonic waves that disrupt the beaded fibers at the junction between the fiber and the beads by operating for 10 s. This process results in sperm-like microrobots comprised of an ellipsoidal bead and a uniform fiber.

### 2.3 SEM characterization

The morphology and geometry of the sperm-like microrobots were investigated by scanning electron microscope (Lyra 3 XM, Tescan, Czech Republic) operating at 5 kV. Furthermore, the chemical composition of the microrobot and the distribution of Fe signals were characterized by energy-dispersive X-ray spectroscopy (EDS) elemental mapping. A 20 nm thick layer of gold was deposited on the microrobots to increase the conductivity for SEM characterizations and EDS mapping.

### 2.4 Magnetization measurements

The magnetic hysteresis loop of beaded fibers was obtained using the embedded vibrating sample magnetometer in a physical property measurement system (9T 2-400K, Quantum Design, Belgium) at room temperature. The applied field ranged from -1000 mT to 1000 mT.

### 2.5 Preparation of Methyl Cellulose (MC) solutions

Methyl Cellulose (MC) (M0512-100G, Sigma Aldrich, The Netherlands) solutions with the w/v concentrations of 0.1% and 0.2% were prepared using the following method. First, 1.5 g of MC was thoroughly dissolved in 150 mL of deionized (DI) water with magnetic stirring for 12 h. The resulting MC solution was filtered twice via suction filtration using a filter with a pore size of 12 μm. This produced a stock MC solution with the w/v concentration of 1%, which was stored in the refrigerator at $4\;{}^{\circ }$C. The 0.1% and 0.2% w/v MC solution were obtained by diluting the 1% w/v MC stock solution with DI water.

### 2.6 Viscosity measurements

The viscosity measurements of the MC solutions were conducted at room temperature using a rheometer (MCR 92, Anton Paar, Austria) with a cone-plate geometry. A shear rate ranging from 10 to 1000 1/s was applied to the MC solution, and the rheometer recorded the resulting shear stress. The viscosities of the mediums can be calculated through the ratio of shear stress to shear rate.

### 2.7 Magnetic actuation and microscopic observation setup

An electromagnetic coil system was built to generate an oscillating magnetic field in a 2D plane. The current applied to two pairs of coils was produced and amplified using the XenusPlus EtherCAT (XE2-230-20, Copley Controls, Canton, USA). This current output system was controlled by a C++—based program. During swimming tests, the oscillating magnetic field with an oscillation angle of 70°and a field strength of 5 mT was generated. The actuation frequency was alternated by controlling the frequency of the output current from XenusPlus EtherCAT. The propulsion of the sperm-like microrobots was recorded using a microscopic observation setup, which consists of a charge-coupled device camera (AVA1000-100GM, Basler AG, Ahrensburg, Germany) and an optical microscope with a 10x objective lens (HI PLAN 10x/0.25 PH, Leica, Germany).

### 2.8 Position acquisition of wave patterns

Our approach combines image processing techniques and custom-written MATLAB scripts to accurately capture and analyze the wave patterns of our sperm-like microrobots. The detailed procedures are described below. First, the pre-processing is conducted on the captured videos of sperm-like microrobots' propulsion using Fiji software. This includes selecting the region of interest, creating reversed images, subtracting the background, and saving the processed frames as a tiff stack for the subsequent processing. In the processed image stack, the head of the microrobot appears as a bright spot owing to the high concentration of magnetic nanoparticles. A threshold is applied to filter the head. For tail tracking, we employ a Gaussian filter to smooth each frame in the stack, followed by tracking the wave patterns using gradient vector flow and active contour models in a customized MATLAB script [25], [26]. Active contours, which are deformable curves, are used to locate the boundaries of the microrobot's tail. The external force, known as gradient vector flow (GVF), is computed as a diffusion of the gradient vectors of a gray-level edge map derived from the image and incorporated into the active contour model. The vector field converges toward the pixels of the tail with the maximum intensity. The active contours are deformed according to the GVF to acquire the positions of the wave patterns, enhancing the tracking precision.

## 3 Results and discussion

### 3.1 Fabrication and magnetic actuation of soft sperm-like microrobots

Sperm cells exhibit planar traveling waves to achieve propulsion using their flexible and slender tail [27]. To create sperm-like microrobots, it is essential to replicate the flexibility and slenderness of sperm tails. Several strategies have been explored to achieve this, such as the use of flagella from microorganisms [28] and the fabrication of soft nanowires [29], [30] or fibers [13]. Specifically, fibers can be fabricated through various methods, including phase separation [31], microfluidic techniques [32], drawing [33], and self-assembly [34]. However, these methods are time-consuming and have constraints on fiber length and material selection. Electrospinning offers several advantages over other methods, such as cost-effectiveness, mass production, and suitability for a wide range of polymers [35].

**Fig. 2:**
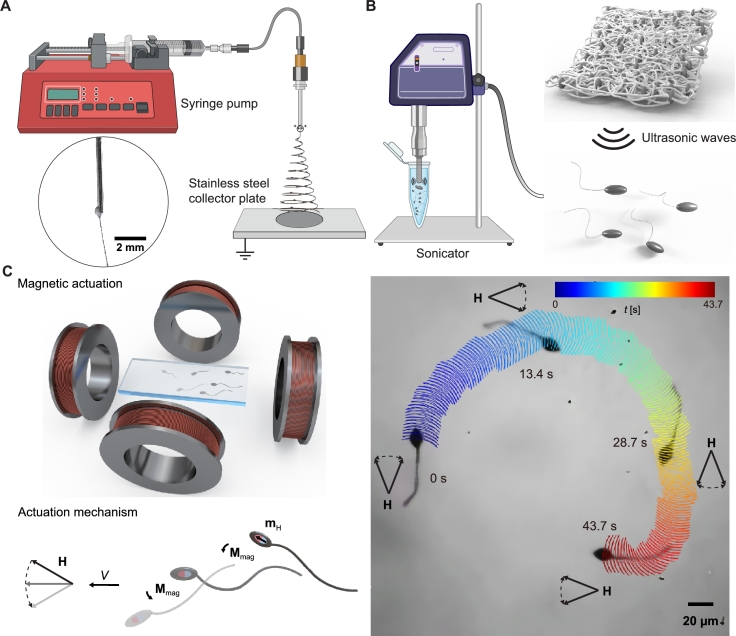
Fabrication and magnetic actuation of sperm-like microrobots. (A) Schematics illustrate the fabrication of beaded fibers using electrospinning. The ejection of fibers from the Taylor cone is depicted in the circular inset. (B) Schematics showcase the ultrasonic breaking of beaded fibers into individual sperm-like microrobots. (C) Upon exposure to an oscillating magnetic field, the head of the sperm-like microrobot experiences a magnetic torque, denoted as **M**_mag_. The continuous oscillation of the head, driven by this torque, enables the tail to generate traveling waves to achieve propulsion. The microscopic image captures the movement and directional change of a sperm-like microrobot in response to the oscillating field, while the colorful curve represents its time-dependent trajectory (blue for earlier times, red for later times).

The soft sperm-like microrobots were fabricated using a customized electrospinning machine and a sonicator, as described in Materials and Methods. Fig. 2A depicts the electrospinning process used to produce beaded fibers, while Fig. 2B illustrates how these fibers are broken up to yield sperm-like microrobots. After sonication cutting, the resulting structures include individual beads, individual fibers, strings of beads, and the desired sperm-like microrobot. Fig. S1 presents the rates of output for all structures, with sperm-like microrobots achieving a yield of 44.7%. These microrobots possess magnetization due to the Fe_3_O_4_ nanoparticles, allowing them to respond to external magnetic fields. The elastic polystyrene fibers ensure tail deformation and facilitate the generation of planar traveling waves. Fig. 2C clarifies the mechanism of magnetic actuation for the sperm-like microrobots. These microrobots achieve planar flagella propulsion when subjected to an in-plane oscillating magnetic field, in contrast to the helical flagellar propulsion observed under an out-of-plane precession magnetic field [29]. The locomotion capability of this torque-driven microrobot is influenced by its magnetic property. The as-prepared sperm-like microrobots undergo subsequent characterization to assess their geometry, chemical composition, and magnetization.

### 3.2 Geometric, chemical, and magnetization characterization

**Fig. 3:**
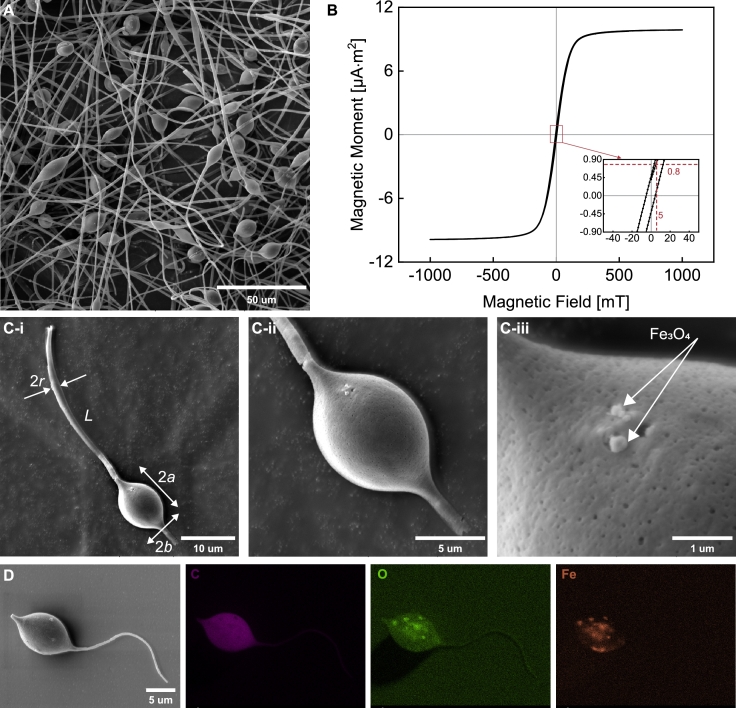
Geometric, chemical, and magnetization characterization of beaded fibers and sperm-like microrobots. (A) Scanning electron microscope (SEM) image of the beaded fibers. (B) Magnetic hysteresis loop of the beaded fibers. The inset shows the generated magnetic moment of the fibers under a magnetic field ranging from -50 mT to 50 mT. A field strength of 5 mT is applied, inducing a magnetic moment of $0.8\;\mu A\cdot m^{2}$ on the fibers, as indicated by the red dashed lines. (C) SEM images are taken of the sperm-like microrobot, including (i) the full microrobot and (ii) (iii) a zoom-in view of its head. The geometrical parameters of the sperm-like microrobot, including the radius of the tail, *r*, the tail length, *L*, the major radius, *a*, and the minor radius, *b*, of the ellipsoidal head, are displayed in (i). (D) Energy-dispersive X-ray spectroscopy (EDS) elemental mapping images of C, O, and Fe in the sperm-like microrobot are shown.

The detailed beaded fibers were observed through SEM characterization. The SEM image presented in Fig. 3A shows the presence of beads that can function as the head of sperm-like microrobots. The emergence of these beads is attributed to the polymer solution's low viscosity and the limited density of surface charges on the droplet [24]. With the inclusion of magnetic nanoparticles, these beaded fibers can generate a magnetic moment when exposed to an external magnetic field. This field further induces a magnetic torque that drives the soft tail to undergo undulations, leading to the planar flagellar propulsion [36]. Fig. 3B illustrates the induced magnetic moment of the beaded fibers under an external field ranging from -1000 mT to 1000 mT. The magnetization response of the beaded fibers varies with the applied field strength and serves as the basis for calculating the maximum magnetic torques acting on the microrobots.

Upon sonication cutting, the beaded fibers were fragmented to produce the sperm-like microrobots. The microrobot's geometrical and chemical properties were characterized using SEM and EDS elemental mapping, respectively. Fig. 3C-i presents the SEM image of a sperm-like microrobot, which consists of a slender tail and an ellipsoidal head. Further examination of the zoomed-in SEM images in Fig. 3C-ii and 3C-iii reveals that some magnetic nanoparticles are located on the surface of the microrobot's head. EDS elemental mapping was performed to assess the distribution of Fe_3_O_4_ within the entire structure of the microrobot. The results show that C, O, and Fe are mainly distributed in the head owing to the larger volume relative to the tail (Fig. 3D). The mapping image of Fe also indicates that Fe_3_O_4_ nanoparticles reside inside the head, rather than merely on its surface. Considering the distribution of Fe_3_O_4_ nanoparticles within the sperm-like microrobot, the magnetic torque exerted on the tail is relatively minor compared to that exerted on its head.

### 3.3 Nondimensional analysis of magneto-ElastoHydrodynamics

The EDS elemental mapping indicates a trivial amount of magnetic nanoparticles on the tail. Consequently, the magnetic torque exerted on the head is considered the sole energy source for actuating the microrobot. In response to an oscillating magnetic field, denoted as **H**, the magnetic head of the sperm-like microrobot becomes magnetized, exhibiting a magnetization per unit volume of $m_{H}$. This results in a magnetic torque, $M_{\mathrm{mag}}$, which is given by

(1) $$M_{\mathrm{mag}}=\mu_{0}vm_{H}\times H\;,$$

where $\mu_{0}$ is the permeability of free space, *v* is the volume of the sperm-like microrobot's head.

As the microrobot moves through a medium, it encounters viscous drag torques, $M_{\mathrm{head}}$ and $M_{\mathrm{tail}}$, on its head and tail, respectively. These viscous drag torques are defined by the analytical function that accounts for the geometry of the microrobot. The sperm-like microrobot features an ellipsoidal head attached to a soft tail, as observed in Fig. 3C-i. The geometrical parameters include the radius of the tail, *r*, the tail length, *L*, the major radius, *a*, and the minor radius, *b*, of the ellipsoidal head. The bending stiffness, *E*, of the tail is calculated by multiplying the electrospun fiber's Young's modulus by the area moment of inertia of the fiber's cross-section. Chwang and Wu [37] derived the viscous drag torque on a prolate ellipsoid, which is utilized to express $M_{\mathrm{head}}$ as follows:

(2) $$\left|M_{\text{head }}\right|=8ab^{2}\pi C_{1}\eta \omega \;.$$

The torque coefficient, $C_{1}$, is associated with the ellipsoid's eccentricity, denoted as $\epsilon =\sqrt{1-{\left(b/a\right)}^{2}}$, and *η* is the viscosity of the surrounding medium. The interaction between medium and tail is described by resistive force theory [38]. The local viscous drag force on the tail depends on its local velocity relative to the medium. Based on the assumption of small amplitude, the small segment on the tail moves with the local velocity normal to the major axis of the head [39], [40]. The viscous drag torque on the tail can be determined via the following equation:

(3) $$\left|M_{\mathrm{tail}}\right|=\int_{0}^{L}\xi_{⊥}xdy/dt\;dx\;,$$

where $\xi_{⊥}$ is the normal drag coefficient of the tail. A sine wave of constant amplitude, $y(x,t)=y_{0}e^{i2\pi (ft-x/\lambda )}$, represents the wave patterns in response to the oscillating magnetic field with the frequency of *f*. The wave variables $y_{0}$ and *λ* are the averaged bending amplitude and wavelength of the wave patterns, respectively.

The bending wave along the tail, which enables the propulsion of the sperm-like microrobot, is achieved through the magneto-elastohydrodynamic coupling between the deformable tail, the external field, and the surrounding medium. Without loss of generality, we conduct a nondimensional analysis of this magneto-elastohydrodynamics. The nondimensional viscous drag torques, $M_{\mathrm{head}}$ and $M_{\mathrm{tail}}$, on the head and the tail are given by,

(4) $$M_{\mathrm{head}}=R_{\mathrm{head}}{\mathrm{Sp}}^{4},\;\;\;M_{\mathrm{tail}}=R_{\mathrm{tail}}{\mathrm{Sp}}^{4}\;,$$

where $R_{\mathrm{head}}$ and $R_{\mathrm{tail}}$ are nondimensional drag coefficients determined by the geometry of the head and the wave patterns, respectively. The nondimensional drag coefficient of the head is given by the analytic function $R_{\mathrm{head}}=8ab^{2}\pi C_{1}\eta /L^{3}\xi_{⊥}$, while that of the tail depends on the wave variables $y_{0}$ and *λ*, expressed as $R_{\mathrm{tail}}=y_{0}\lambda /2\pi L^{2}$. In addition, the nondimensional magnetic torque, $M_{\mathrm{mag}}$, is expressed by:

(5) $$M_{\mathrm{mag}}=M\left|{\overset{ˆ}{m}}_{H}\times \overset{ˆ}{H}\right|\;,$$

where $M=\mu_{0}v\left|m_{H}\right|\left|H\right|L/E$ is the magnetic number, which denotes the ratio between the magnetic and elastic torques, and the ‘hat’ symbol represents the normalized form of the variable. The sperm number Sp, defined as the equation $\mathrm{Sp}=L{\left(\xi_{⊥}\omega /E\right)}^{1/4}$, characterizes the relative importance of elastic force to viscous drag force, representing the floppiness of the tail [41]. It can be determined by constructing a nondimensional torque balance equation between Equations (4) and (5):

(6) $$\mathrm{Sp}=\sqrt[{4}]{\frac{M{\overset{ˆ}{m}}_{H}\times \overset{ˆ}{H}}{R_{\mathrm{head}}+R_{\mathrm{tail}}}}\;,$$

When the angular frequency, represented by *ω*, of the oscillating magnetic field matches the step-out angular frequency, denoted as $\omega_{\mathrm{so}}$, the cumulative viscous drag torque equals the maximum magnetic torque. The step-out frequency, $f_{\mathrm{so}}=\omega_{\mathrm{so}}/2\pi$, can be obtained by studying the maximum magnetic torque and the viscous drag torque. The maximum nondimensional magnetic torque, $\mathrm{Max}(M_{\mathrm{mag}})$, on the soft-magnetic ellipsoidal body, is as follows [42]:

(7) $$\mathrm{Max}(M_{\mathrm{mag}})=\frac{\mu_{0}v\left|n_{\mathrm{rad}}-n_{\mathrm{axi}}\right|{\left|m_{H}\right|}^{2}L}{2E}\;,$$

where the demagnetization factors, $n_{\mathrm{rad}}$ and $n_{\mathrm{axi}}$, are along all radial directions and the major axis of the ellipsoidal body, respectively. Substituting Equation (7) to Equations (6), the sperm number ${\mathrm{Sp}}_{\mathrm{so}}$ at the step-out frequency is obtained as:

(8) $$Sp_{\mathrm{so}}=\sqrt[{4}]{\frac{\mu_{0}v\left|n_{\mathrm{rad}}-n_{\mathrm{axi}}\right|{\left|m_{H}\right|}^{2}L}{2E(R_{\mathrm{head}}+R_{\mathrm{tail}})}}\;.$$

Equation (8) reveals that the sperm number at the step-out frequency depends on the magnetization, the geometry of the sperm-like microrobots, the viscosity of the medium, and the wave patterns of the tail.

### 3.4 Derivation of the equation for step-out frequency and velocity at step-out frequency

Combining Equation (8) with the definition of the sperm number, we can derive the step-out angular frequency, $\omega_{\mathrm{so}}$, as follows:

(9) $$\omega_{\mathrm{so}}=\frac{\mu_{0}v\left|n_{\mathrm{rad}}-n_{\mathrm{axi}}\right|{\left|m_{H}\right|}^{2}}{2(8ab^{2}\pi C_{1}\eta +y_{0}\lambda L\xi_{⊥}/2\pi )}\;,$$

where wave variables $y_{0}$ and *λ* are extracted according to experimentally observed wave patterns. The sperm-like microrobots experience zero magnetic force in the gradient-free field. Their propulsive thrust comes from the transmission of transverse waves along the tail [43], which needs to counteract the viscous drag force on the head. Consequently, we can establish the following force-balance equation to determine the swimming velocities, denoted as *V*, of sperm-like microrobots,

(10) $$\int_{0}^{L}\frac{(\xi_{⊥}-\xi_{∥})(\mathrm{dy}/\mathrm{dt})\frac{dy}{\;dx}-V\left(\xi_{∥}+\xi_{⊥}{\left(\frac{dy}{\;dx}\right)}^{2}\right)}{1+{\left(\frac{dy}{\;dx}\right)}^{2}}dx=6\pi \eta aC_{2}V\;,$$

where $\xi_{∥}$ is the tangent drag coefficient of the tail, and $C_{2}$ is the nondimensional drag coefficient that depends on the dimensions of the sperm-like microrobot's ellipsoidal head. Likewise, the swimming velocity is influenced by the wave patterns of the microrobot. Variables used in the calculations of the step-out frequency and swimming velocity are indicated in Table S1.

### 3.5 Analysis of wave patterns observed at step-out frequency

**Fig. 4:**
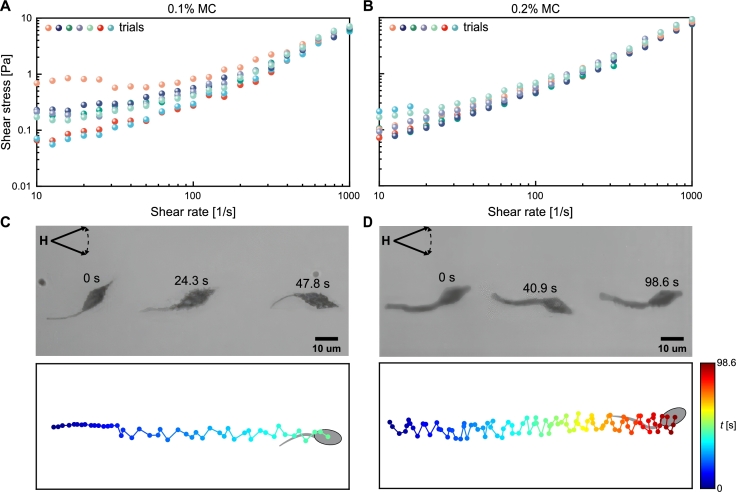
Rheological behavior of Methyl Cellulose (MC) solutions at varying w/v% concentrations and the propulsion of sperm-like microrobots within these mediums. Viscosity measurement of (A) 0.1% and (B) 0.2% MC solutions over seven trials, with each trial differentiated by the symbols with different colors. The average viscosities for 0.1% and 0.2% MC solutions across seven trials are 4.4 mPa ⋅ s and 5.7 mPa ⋅ s, respectively. Sequential frames capture the microrobots' propulsion over the same distance in (C) 0.1% and (D) 0.2% MC solutions and their corresponding time-dependent trajectories on the head are depicted (blue for earlier times, red for later times).

To validate our theoretical model, we performed swimming tests of the sperm-like microrobots in DI water, 0.1% w/v, 0.2% w/v MC solutions to investigate their step-out frequencies. The viscosities of these mediums were measured via a rheometer. Fig. 4A and 4B shows the measured shear stress at different shear rates for the two MC concentrations (see Material and Methods). The results indicate that both MC solutions exhibit constant viscosities despite changes in shear rate. In these two MC solutions, the sperm-like microrobots exhibit distinct swimming velocities. Their propulsion and corresponding trajectories on the head are shown in Fig. 4C and 4D. We exclusively consider the head's displacement along the oscillating axis of the field for evaluating the swimming performance of the sperm microrobot. In the 0.1% MC solution, the sperm-like microrobot moves around 96 μm in a period of 47.8 s, compared to 98.6 s in the 0.2% MC solution. The decrease in the velocity can be attributed to the increasing viscous drag force within the more viscous medium. Moreover, the viscosity is likely to impact the wave patterns.

**Fig. 5:**
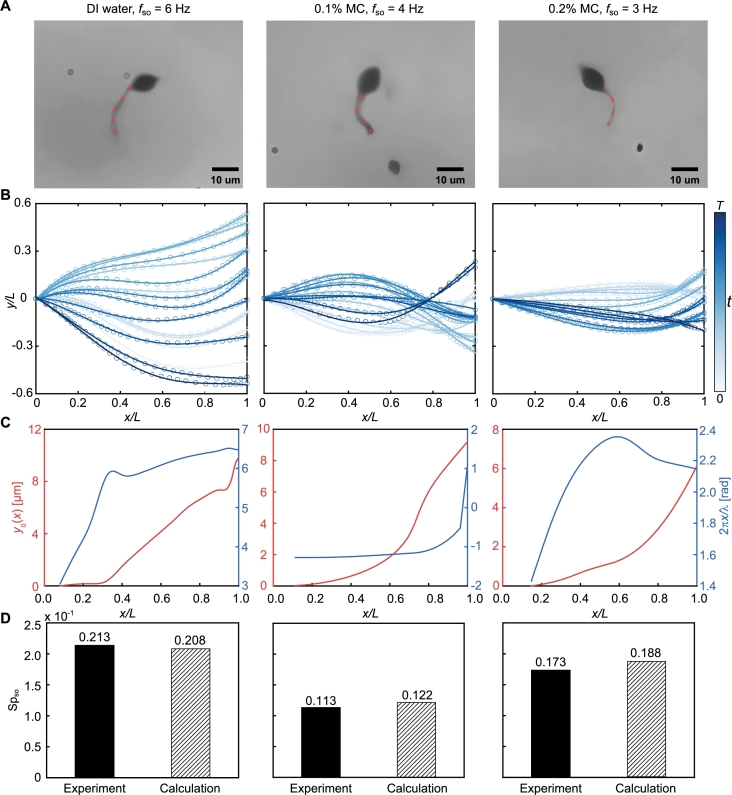
Observation and analysis of sperm-like microrobots' wave patterns at their step-out frequencies in various mediums. (A) Tail's deformation of sperm-like microrobots in deionized (DI) water, 0.1%, and 0.2% MC solutions at their step-out frequencies. The red dashed line describes the centerline of the bending tail. (B) The transverse displacement *y*(*x*,*t*) is determined from the tail deformation throughout one entire beat cycle, with the circles as marked points along the tails connected by polynomial curves (darker curves for later times). *T* is the time for one beat cycle. (C) Time-averaged bending amplitude, *y*_0_(*x*), and the phase angle, 2*πx*/*λ*, are extracted from the observed wave patterns in DI water, 0.1%, and 0.2% MC solutions. (D) Experimental and calculated sperm number at the step-out frequency. The calculated sperm number at the step-out frequency is determined by substituting the extracted wave variables into Equation (8).

The analysis of these wave patterns is undertaken using nonlinear curve fitting, a technique employed to study wave patterns of living spermatozoa [44], [45]. The coordinates of the tail's deformation of sperm-like microrobots were obtained from the captured videos (see Materials and Methods). Fig. 5A shows the propulsion of the sperm-like microrobots in DI water, 0.1% MC, and 0.2% MC solutions, with the red dashed line profiling the centerline of the tail. The tail deformation over one complete beat cycle is illustrated in Fig. 5B. In all cases, the bending wave initiates from the proximal end and propagates toward the distal end. As the viscosity increases, the transverse displacement of the bending wave decreases, which can be attributed to the increased viscous drag force. The wave variables $y_{0}$ and *λ* are obtained by analyzing the extracted curves using the nonlinear curve fitting method, as shown in Fig. 5C. The time-averaged bending amplitude, $y_{0}(x)$, exhibits an increasing trend that corresponds to the increasing amplitude along the tail (refer to Fig. 5B). Similarly, the phase angle, 2πx/λ, shows a rise along the tail, which reveals that the planar waves propagate from head to tail and, in turn, lead to the propulsion from tail to head. Based on the extracted wave variables, we can calculate the sperm number at the step-out frequency using Equation (8). Fig. 5D shows the quantitative agreement between the experimental and the calculated sperm number at the step-out frequency, confirming the validity of our proposed model.

### 3.6 Analysis of wave patterns observed without exceeding step-out frequency

Wave patterns of living spermatozoa undergo noticeable variations in response to changes in the beat frequency. These variations might result from an alteration in the viscosity of the surrounding medium [46], [47], the external force [48], or chemoattractant molecules [49], [50]. Unlike spermatozoa, which deform their flagella through the coordinated cooperation of dynein motors, sperm-like microrobots passively undulate their tails via magnetic torque. Further investigation is needed to understand the effect of actuation frequency on the wave patterns exhibited by sperm-like microrobots.

**Fig. 6:**
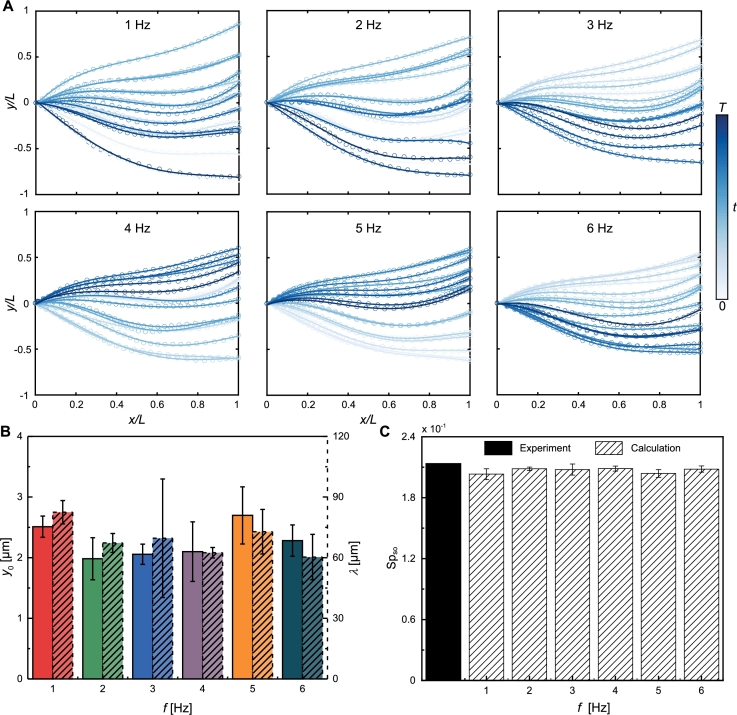
Analysis of the sperm-like microrobot's wave patterns in DI water. (A) The wave patterns of the sperm-like microrobots are displayed at actuation frequencies ranging from 1 Hz to 6 Hz (6 Hz is the step-out frequency in this trial. Darker curves for later times). (B) Average bending amplitude *y*_0_ and wavelength *λ* versus different actuation frequencies. (C) The solid black bar is the experimental sperm number, Sp_so_, at the step-out frequency. The bars filled with diagonal lines represent the calculated Sp_so_, obtained by substituting the wave variables extracted from 1 Hz to 6 Hz into Equation (8).

**Fig. 7:**
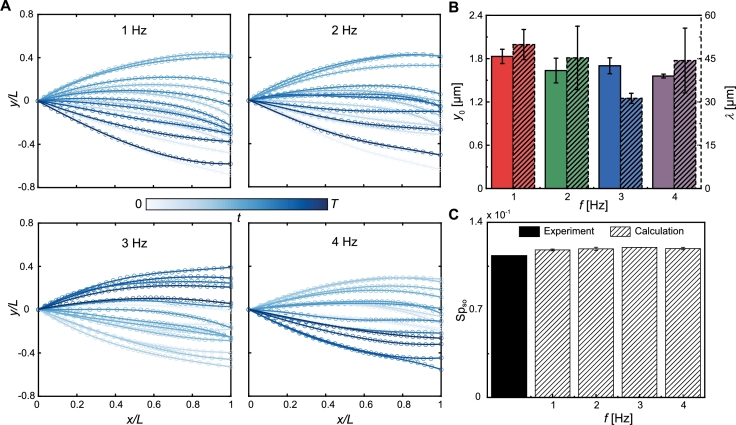
Analysis of the sperm-like microrobot's wave patterns in 0.1% MC solution. (A) The wave patterns of the sperm-like microrobots are displayed at actuation frequencies ranging from 1 Hz to 4 Hz (4 Hz is the step-out frequency in this trial. Darker curves for later times). (B) Average bending amplitude *y*_0_ and wavelength *λ* versus different actuation frequencies. (C) The solid black bar is the experimental sperm number, Sp_so_, at the step-out frequency. The bars filled with diagonal lines represent the calculated Sp_so_, obtained by substituting the wave variables extracted from 1 Hz to 4 Hz into Equation (8).

**Fig. 8:**
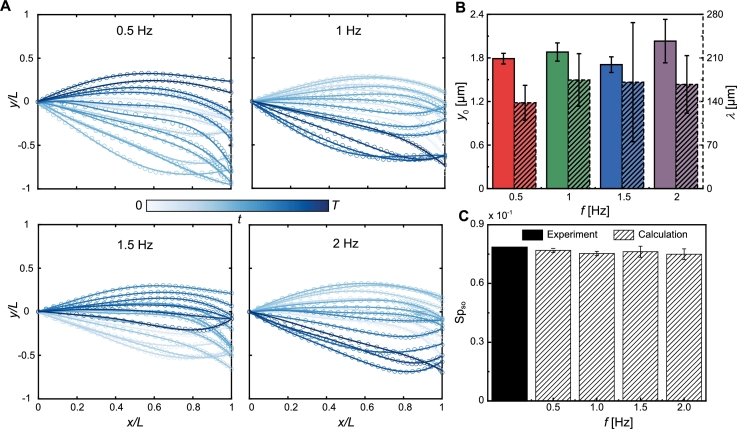
Analysis of the sperm-like microrobot's wave patterns in 0.2% MC solution. (A) The wave patterns of the sperm-like microrobots are displayed at actuation frequencies ranging from 0.5 Hz to 2 Hz (2 Hz is the step-out frequency in this trial. Darker curves for later times). (B) Average bending amplitude *y*_0_ and wavelength *λ* versus different actuation frequencies. (C) The solid black bar is the experimental sperm number, Sp_so_, at the step-out frequency. The bars filled with diagonal lines represent the calculated Sp_so_, obtained by substituting the wave variables extracted from 0.5 Hz to 2 Hz into Equation (8).

The wave patterns of the sperm-like microrobot in DI water, ranging from 1 Hz to 6 Hz, are illustrated in Fig. 6A. The frequency of 6 Hz corresponds to the step-out frequency in the trial. The wave variables for each frequency, extracted in Fig. 6B, align with the observed slight change in the wave patterns as the frequency increases. These slightly changed wave patterns suggest that the undulations of the sperm microrobot remain synchronized with the external field across this range of actuation frequencies. The calculated sperm number closely matches the experimentally measured value at the step-out frequency (Fig. 6C). We also examined the wave patterns of the sperm-like microrobots below the step-out frequency in 0.1% and 0.2% MC solutions, as shown in Figs. 7A and 8A. The wave variables for these mediums are extracted and plotted against actuation frequencies (Figs. 7B and 8B). Minor variations in the wave variables across frequencies introduce slight deviations between the calculated and experimental sperm numbers at the step-out frequency (Figs. 7C and 8C). The precise prediction of the sperm number at the step-out frequency is attributed to the near-constant nondimensional drag coefficients across actuation frequencies. Our findings emphasize that step-out frequencies of the magnetically actuated soft sperm-like microrobots can be theoretically calculated after analyzing their wave patterns at any frequency below the step-out threshold. This approach simplifies the current way of determining the step-out frequencies of sperm-like microrobots by measuring their frequency responses across a wide range of frequencies.

Our theoretical model can accurately predict the step-out frequency of the sperm-like microrobot by analyzing the wave patterns observed at any frequency without exceeding the step-out threshold. This feature enhances the utility of our model, which is supported by theoretical and empirical evidence. Equation (9) indicates that the step-out frequency depends on the wave variables $y_{0}$ and *λ*. During oscillating actuation, the head oscillates with the field, leading to the planar undulation of the flexible tail. The amplitude of the head's oscillation is determined solely by the amplitude of the external field, provided that the head of the microrobot can synchronously follow the external field. As long as the actuation frequency is below the step-out frequency, the head maintains a constant oscillating amplitude, resulting in only slight variations in wave patterns across different frequencies. Our experimental results, presented in Figs. 6B, 7B, and 8B, demonstrate these minor variations in wave variables at frequencies below the step-out threshold. This observed behavior may also be relevant to the soft microrobots consisting of a magnetic rigid component at one end connected to a non-magnetic soft thin segment. When such a microrobot oscillates synchronously with the external field at a constant amplitude, its wave patterns exhibit slight variations across different frequencies before the step-out threshold. Our findings are not limited to our specific sperm-like microrobots but are likely applicable to this broader class of soft microrobots, enhancing the relevance and utility of our theoretical model.

### 3.7 Model validation and guidance

**Fig. 9:**
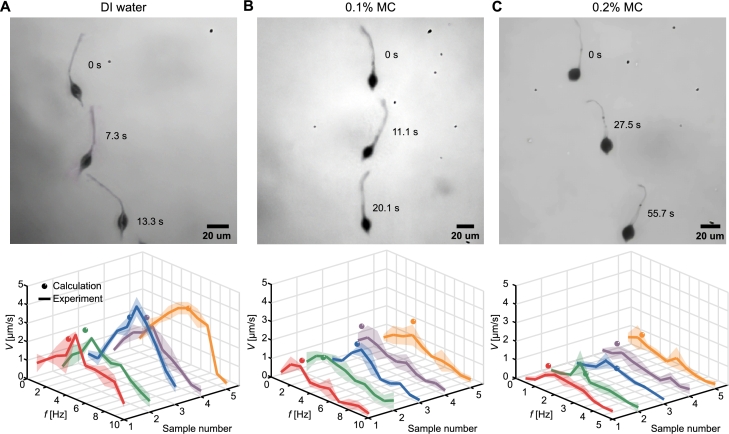
Comparison between experimental and calculated results on the swimming performance of sperm-like microrobots in (A) DI water, (B) 0.1% MC, and (C) 0.2% MC solutions. The upper images illustrate the sequence of the microrobot's movement in DI water, 0.1% MC, and 0.2% MC solutions. The lower plots show frequency response and theoretical calculation of sperm-like microrobots in these mediums. The solid lines denote the averaged velocity of five sperm-like microrobots across a range of actuation frequencies, with the error band representing the standard deviation. The sphere symbols represent the theoretical calculation on the step-out frequency and the maximum velocity derived using Equations (9) and (10).

After conducting the requisite characterizations, we obtained the variables required for Equations (9) and (10). These equations are utilized to determine the step-out frequency and the maximum velocity achieved by the sperm-like microrobot at this frequency. The sperm-like microrobots can propel themselves by generating traveling waves under an oscillating magnetic field (see upper images in Fig. 9 and Movie S1). The swimming velocity of each sperm-like microrobot is measured within a frequency range of 1-10 Hz in DI water, 0.1% MC solution, and a range of 0.5-5 Hz in 0.2% MC solution, as depicted in the lower plots of Fig. 9. Note that the maximum velocity of the sperm-like microrobot with a tail length of 15 μm can reach 4.4 μm/s, approximately 0.3 body lengths per second. The velocities of sperm-like microrobots initially increase with the frequency until the step-out frequency, where they exhibit the maximum velocities. The step-out frequencies of sperm-like microrobots in DI water are in the range of 4-6 Hz (Fig. 9A). The observed discrepancy is attributed to the varying amount of the magnetic nanoparticles in each sperm-like microrobot. In 0.1% MC solution, the step-out frequencies decrease to the range of 3-5 Hz (Fig. 9B), while the step-out frequencies reduce further to the range of 2-3 Hz in 0.2% MC solution (Fig. 9C). The decrease in the step-out frequency with the increased viscosity of the medium corresponds with Equation (9). Moreover, the velocities of sperm-like microrobots decrease when they encounter larger viscous drag forces in mediums with higher viscosities. The wave variables, geometrical parameters, the viscosities of the mediums, and the magnetization obtained at the above sections are substituted into Equations (9) and (10), yielding the predicted values for the step-out frequency, $f_{\mathrm{so}}$, and the swimming velocity, *V*, of each sperm-like microrobot. The theoretical results are in good agreement with the experimental results, substantiating the robustness and reliability of the proposed model.

Actuated under the predicted step-out frequency, magnetically actuated soft microrobots with low step-out frequencies can still exhibit precise motion and execute complicated tasks. The proposed model can help prevent situations where an excessively high actuation frequency undermines the precision of a microrobot's motion. Additionally, the step-out frequency directly impacts the maximum swimming velocity of soft microrobots, as this velocity is proportional to the step-out frequency. The calculation of the maximum velocity allows us to analyze the microrobots' swimming capability, which is crucial for tailoring the microrobots to specific tasks. For instance, fast movement enables the microrobots to promptly reach specific locations when executing targeted delivery applications. Conversely, slow and precise movements are required for handling delicate or intricate operations in micromanipulation tasks. Furthermore, accurately predicting the maximum velocity is critical for effective navigation and trajectory planning of magnetic microrobots, especially in open-loop control systems, where position feedback is unavailable. To achieve a high step-out frequency, inducing a large magnetic moment on the microrobot is a viable approach. This magnetic moment can be enhanced through methods such as increasing the field strength, raising the volume fraction of magnetic material, and incorporating hard magnetic material during fabrication. Although augmenting the head's geometry can heighten the volume fraction of magnetic material, it also inadvertently elevates the viscous drag on the microrobot, resulting in a decrease in the step-out frequency. Therefore, careful consideration of the microrobot's geometry is essential to strike an optimal balance between magnetic moment enhancement and minimized viscous drag effects. To conclude, accurately predicting step-out frequencies and the maximum velocities is pivotal for ensuring the precision and efficiency of soft microrobots in biomedical applications.

## 4 Conclusion

In this paper, we have proposed an analytic model for determining the step-out frequency of magnetically actuated soft microrobots. This model enhances our understanding of the relationship between the step-out frequency and various factors, including the microrobot's geometry, magnetization, wave patterns, and the viscosity of the surrounding medium. The experimental results not only validate the accuracy of our proposed analytic model for the step-out frequency but also reveal the independence of wave patterns from actuation frequency, in contrast to natural sperm cells. This distinct behavior allows our analytic model to accurately predict the step-out frequency of the sperm-like microrobot by evaluating the observed wave patterns at any frequency below the step-out threshold. The accurate prediction of the step-out frequency using our model has important implications for the control and navigation of individual microrobots as well as swarms of microrobots. The synchronized motion of individuals is crucial for executing complex tasks and maneuvers as it enables precise navigation and predictable behavior of the microrobot. Furthermore, the model can facilitate selective control within a swarm by allowing different microrobots to be actuated at frequencies below their respective step-out frequencies. This selective control enables the targeted and coordinated movement of individual microrobots within the swarm.

While our current model offers valuable insights into the control of sperm-like microrobots, several limitations remain to be addressed in future research. These limitations include three-dimensional navigation of the sperm-like microrobots, model optimization to study the step-out frequency during this navigation, real-time tracking of the wave patterns, and the optimized magnetization profile for enhanced swimming efficiency. Considering that sperm-like microrobots with helical flagellar waves exhibit higher swimming efficiency than those with planar flagellar waves and resemble the motion of natural sperm cells, it is crucial to develop a theoretical model for determining the step-out frequency of these microrobots under a precession magnetic field. This involves the tracking of wave patterns in three dimensions. Moreover, the real-time analysis of wave patterns should be combined with the actuation strategies to determine the appropriate actuation frequency, which depends on the application scenarios, such as synchronized and efficient control of individuals and selective control in a swarm. Additionally, alternative designs, such as sperm-like microrobots with a magnetic soft tail, might exhibit higher step-out frequencies and swimming efficiencies. To explore these possibilities, the current model should be upgraded to incorporate the magnetic torque on the tail. The upgraded model would enable the formulation of an optimization problem to study the ideal magnetization profile along the tail of the microrobot, aiming for achieving optimal step-out frequency or swimming velocity. The inclusion of magnetization along the tail will cause the axis of rotation to vary from the head's centroid, impacting the viscous drag torque on the head. Generalizing the model to account for rotation about any arbitrary axis is meaningful yet challenging. The analytical form for the viscous drag torque when considering arbitrary rotation axes is complex and unknown. Numerical calculations might offer a way to determine this viscous drag torque when the ellipsoid rotates about any axis. These calculations would allow for understanding the effects of different rotation axes on the viscous drag torque and further determining the step-out frequency of the sperm-like microrobot with a magnetic tail.

## CRediT authorship contribution statement

**Zihan Wang:** Writing – original draft, Validation, Investigation, Formal analysis. **Wenjian Li:** Writing – review & editing, Investigation. **Anke Klingner:** Writing – review & editing, Software. **Yutao Pei:** Writing – review & editing. **Sarthak Misra:** Writing – review & editing. **Islam S.M. Khalil:** Writing – review & editing, Supervision, Project administration, Conceptualization.

## Declaration of Competing Interest

The authors declare that they have no known competing financial interests or personal relationships that could have appeared to influence the work reported in this paper.

## Data Availability

Data will be made available on request.

## Appendix A Supplementary material

MMC Supplementary information about the geometry and drag coefficients of the microrobots. MMC

Video Movement of magnetically actuated soft sperm-like microrobots in deionized water, 0.1%, and 0.2% Methyl Cellulose solutions. Video
