# Supplementary material for: Magnetic control of soft microrobots near step-out frequency: Characterization and analysis
Source: Comput Struct Biotechnol J. 2024 Aug 30;25:165–76. doi: 10.1016/j.csbj.2024.08.022 (PMC11630648; doi:10.1016/j.csbj.2024.08.022)
Supplement: MMC — Supplementary information about the geometry and drag coefficients of the microrobots. [file mmc1.pdf]

Supplementary Materials
Magnetic Control of Soft Microrobots Near Step-Out Frequency: Characterization and Analysis

Zihan Wang[a,*], Wenjian Li[b], Anke Klingner[c], Yutao Pei[b], Sarthak Misra[a,d], Islam S. M. Khalil[d,*]

[a] *Department of Biomaterials and Biomedical Technology, University of Groningen and University Medical Center Groningen, Groningen, 9713 GZ, The Netherlands*
[b] *Department of Advanced Production Engineering, Engineering and Technology Institute Groningen, University of Groningen, Groningen, 9747 AG, The Netherlands*
[c] *Department of Physics, The German University in Cairo, New Cairo, 11835, Egypt*
[d] *Department of Biomechanical Engineering, University of Twente, Enschede, 7500 AE, The Netherlands*

This PDF file includes:

Figure S1
Table S1
Description of Movie S1

*Corresponding author
Email addresses: z.wang04@umcg.nl (Zihan Wang), i.s.m.khalil@utwente.nl (Islam S. M. Khalil)

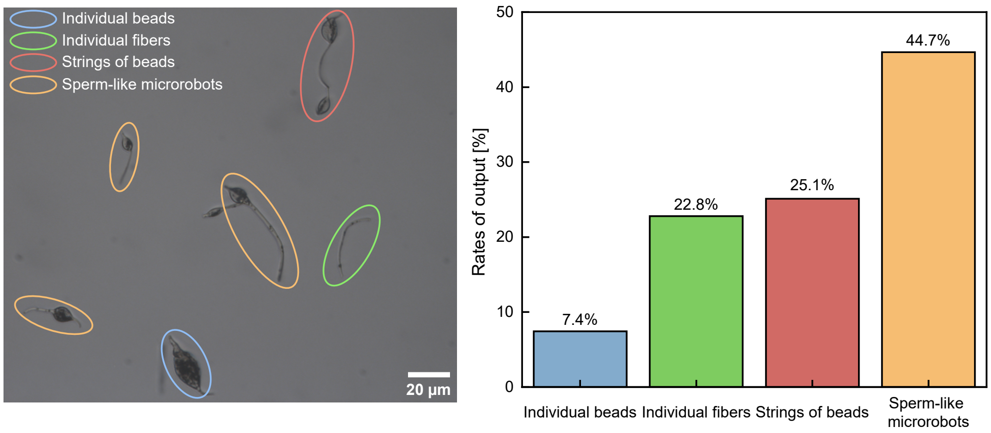

Figure S1: Various structures after sonication cutting include individual beads, individual fibers, strings of beads, and sperm-like microrobots. The rates of output for each structure are statistically analyzed, with a sample size of $n = 200$.

Table S1: Values, calculations, or measurement methods of the variables used in the calculations.

tail length L, tail radius r, the major radius a and minor radius b of the head	These variables are measured from scanning electron microscopy (SEM) and optical micrographs.
volume of the head v	$v = \frac{4}{3}\pi ab^2$
the permeability of free space μ_0, the magnetization of the head $\mathbf{m_H}$, the bending stiffness of the tail E	$\mu_0 = 4\pi \times 10^{-7}\ \mathrm{T \cdot m/A}$ $\lvert\mathbf{m_H}\rvert = 2.96 \times 10^3\ \mathrm{Am} \quad E = 0.58\ \mathrm{GPa}$
demagnetization factors n_{rad} and n_{axi} along all radial directions and major axis of the ellipsoidal head, the ratio R of major radius to the minor radius	$n_{\mathrm{axi}} = \frac{1}{R^2-1}\left(\frac{R}{2\sqrt{R^2-1}} \ln\left(\frac{R+\sqrt{R^2-1}}{R-\sqrt{R^2-1}}\right) - 1\right)$ $n_{\mathrm{axi}} + 2n_{\mathrm{rad}} = 1 \qquad R = a/b$
torque coefficient C_1 and drag coefficient C_2, the ellipsoidal head's eccentricity	$C_1 = \frac{4}{3}\epsilon^3\left(\frac{2-\epsilon^2}{1-\epsilon^2}\right)\left[-2\epsilon + (1+\epsilon^2)\ln\frac{1+\epsilon}{1-\epsilon}\right]^{-1}$ $C_2 = \frac{8}{3}\epsilon^3\left[-2\epsilon + (1+\epsilon^2)\ln\frac{1+\epsilon}{1\epsilon}\right]^{-1}$ $\epsilon = \sqrt{1-(b/a)^2}$
viscosity of the medium η	This variable is measured using a rheometer, The viscosities of deionized (DI) water, 0.1% methylcellulose (MC), and 0.2% MC solution are 1 mPa·s, 4.4 mPa·s, and 5.7 mPa·s, respectively.
normal drag coefficient $\xi_\perp$ and tangent drag coefficient $\xi_\parallel$ of the tail	$\xi_\perp = \frac{4\pi\eta}{\ln(L/r)+0.193} \quad \xi_\parallel = \frac{2\pi\eta}{\ln(L/r)-0.807}$
wave variables y_0 and λ	Wave variables are determined via the nonlinear curve fitting method, as introduced in *section 3.5*. They are dependent on the wave patterns of sperm-like microrobots.

Movie S1. Movement of magnetically actuated soft sperm-like microrobots in deionized (DI) water, 0.1%, and 0.2% Methyl Cellulose (MC) solutions.
This video shows the movement of sperm-like microrobots through planar flagellar propulsion under an in-plane oscillating magnetic field with a strength of 5 mT.
